# Chylomicrons Promote Intestinal Absorption and Systemic Dissemination of Dietary Antigen (Ovalbumin) in Mice

**DOI:** 10.1371/journal.pone.0008442

**Published:** 2009-12-24

**Authors:** Yuehui Wang, Sarbani Ghoshal, Martin Ward, Willem de Villiers, Jerold Woodward, Erik Eckhardt

**Affiliations:** 1 Department of Internal Medicine, University of Kentucky, Lexington, Kentucky, United States of America; 2 Graduate Center for Nutritional Sciences, University of Kentucky, Lexington, Kentucky, United States of America; 3 Department of Microbiology, Immunology and Molecular Medicine, University of Kentucky, Lexington, Kentucky, United States of America; Emory University, United States of America

## Abstract

**Background:**

A small fraction of dietary protein survives enzymatic degradation and is absorbed in potentially antigenic form. This can trigger inflammatory responses in patients with celiac disease or food allergies, but typically induces systemic immunological tolerance (oral tolerance). At present it is not clear how dietary antigens are absorbed. Most food staples, including those with common antigens such as peanuts, eggs, and milk, contain long-chain triglycerides (LCT), which stimulate mesenteric lymph flux and postprandial transport of chylomicrons through mesenteric lymph nodes (MLN) and blood. Most dietary antigens, like ovalbumin (OVA), are emulsifiers, predicting affinity for chylomicrons. We hypothesized that chylomicron formation promotes intestinal absorption and systemic dissemination of dietary antigens.

**Methodology/Principal Findings:**

Absorption of OVA into MLN and blood was significantly enhanced when OVA was gavaged into fasted mice together with LCT compared with medium-chain triglycerides (MCT), which do not stimulate chylomicron formation. The effect of LCT was blocked by the addition of an inhibitor of chylomicron secretion, Pluronic L-81. Adoptively transferred OVA-specific DO11.10 T-cells proliferated more extensively in peripheral lymph nodes when OVA was gavaged with LCT than with MCT or LCT plus Pluronic L-81, suggesting that dietary OVA is systemically disseminated. Most dietary OVA in plasma was associated with chylomicrons, suggesting that these particles mediate systemic antigen dissemination. Intestinal-epithelial CaCo-2 cells secreted more cell-associated, exogenous OVA when stimulated with oleic-acid than with butyric acid, and the secreted OVA appeared to be associated with chylomicrons.

**Conclusions/Significance:**

Postprandial chylomicron formation profoundly affects absorption and systemic dissemination of dietary antigens. The fat content of a meal may affect immune responses to dietary antigens by modulating antigen absorption and transport.

## Introduction

Our diet contains many potentially antigenic proteins. The majority of these are enzymatically degraded, but a small fraction survives and enters the body through as yet largely unknown mechanisms. In healthy individuals, this usually leads to systemic immunological tolerance (“oral tolerance”), but in sensitized individuals, absorption can cause significant morbidity, such as with celiac disease or food allergies. Intestinal antigen absorption thus is highly important in health and disease, but knowledge of the mechanisms is limited. Mechanisms involved in sampling of gut micro-organisms, such as transcytosis of particulate matter across epithelial microfold-cells [1] or protrusion of dendritic cell extensions across the intestinal epithelium [2] are not known to be involved in the absorption of soluble dietary antigens, and paracellular leakage across the epithelium is unlikely to occur in healthy individuals due to the presence of strong tight junctions [3], [4].

Recently, it was found that intestinal epithelial cells (IEC) internalize dietary antigens, such as egg-hen albumin (ovalbumin; “OVA”), at the apical surface and secrete part of the antigens from the basolateral surface in association with vesicles (exosomes) [5]–[7]. This would allow dendritic cells in the lamina propria to sample the antigens, by internalizing the exosomes. The physiological relevance of exosomal antigen absorption is unclear, and it is not known whether this mechanism is regulated by food intake.

IEC secrete a distinct class of large lipoprotein particles in the postprandial state, the chylomicrons [8], [9]. These particles enable transport of intestinally absorbed, poorly soluble long-chain triglycerides (LCT) from the gut to other tissues. Dietary short- or medium-chain triglycerides (MCT) do not require chylomicron formation for their absorption and do not stimulate chylomicron secretion. Interestingly, chylomicrons are drained via intestinal lymphatics and are therefore transported through mesenteric lymph nodes (MLN), before reaching the bloodstream at the level of the left-subclavean vein. Postprandial chylomicron formation stimulates mesenteric T-cell proliferation [10], [11], which has typically been attributed to mitogenic effects of fatty acids. We have recently demonstrated that chylomicron formation promotes intestinal absorption of bacterial lipopolysaccharides (LPS) [12], and that LPS is transported through the MLN. LPS transport likely occurred in association with chylomicrons, which are known to bind LPS [13], [14]. Interestingly, lipoproteins with structural similarity to chylomicrons, HDL, bind many different proteins and peptides [15], [16], and dietary antigens, such as OVA, peanut albumins, and milk caseins, have emulsifying properties [17]–[20], which suggests that protein antigens may also have affinity for chylomicrons.

We therefore hypothesized that intestinal absorption of dietary OVA is enhanced if the OVA is ingested in the context of chylomicron formation. We observed indeed that dietary LCT enhanced OVA absorption compared with MCT, and that the effect of LCT was entirely sensitive to an inhibitor of chylomicron secretion, Pluronic L-81 [21]. We also observed that plasma chylomicrons contained most of the dietary OVA, suggesting that these particles mediated systemic antigen dissemination. This was reflected by strongly enhanced antigen specific proliferation of T-cells in peripheral, non-gut draining lymph nodes. Lastly, in vitro studies with CaCo-2 cells showed that chylomicron secretion correlated with basolateral OVA secretion, suggesting that this mechanism may contribute to antigen absorption. Collectively, these data reveal a novel absorption mechanism for dietary protein antigens, which may profoundly affect immune responses to dietary antigens.

## Results

### Dietary LCT promote intestinal absorption of dietary OVA in a chylomicron-dependent manner

**Figure 1:**
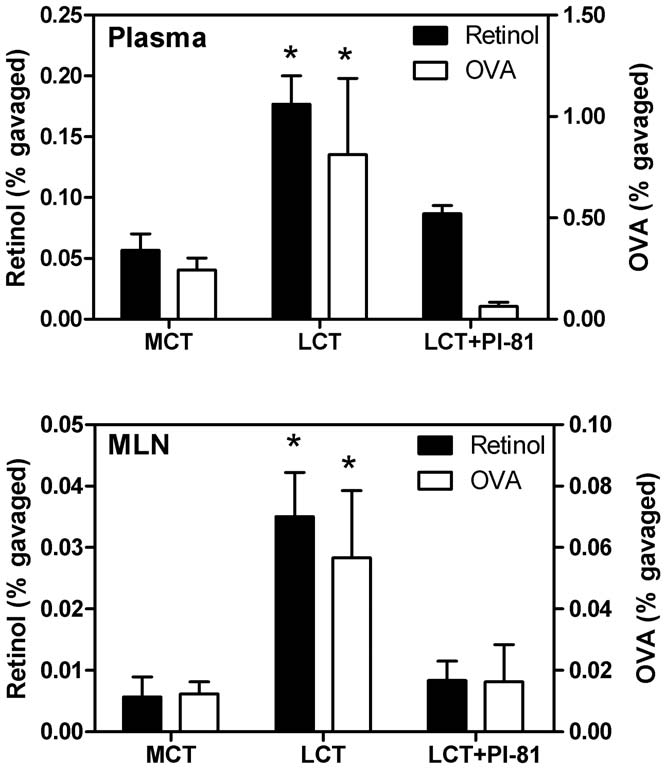
Chylomicron formation promotes intestinal OVA absorption. Fasted mice were gavaged with a dispersion of 0.05 ml ^125^I-labeled OVA (black bars) in PBS plus 0.15 ml of either LCT, MCT, or LCT plus 6 µl of Pluronic L-81 (Pl-81). Radioactivity in the entire plasma per mouse (top panel) and in pooled MLN per mouse (bottom panel) was measured 90 minutes later. Another group of mice was gavaged with identical solutions, except that ^125^I-OVA was replaced with [^3^H]-retinol (white bars). Shown are averages±S.D. of 4 mice per experimental group; * indicates statistically significant differences between feeding groups (P<0.05; ANOVA, Bonferroni's posthoc analysis). The figure shows a representative outcome of two repeats.

**Figure 2:**
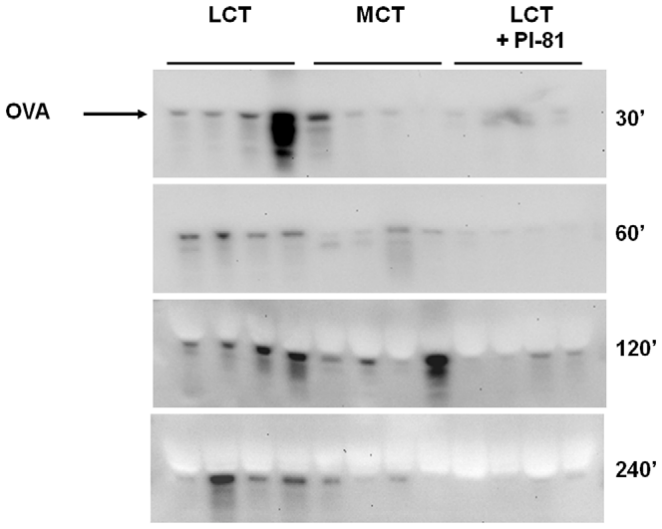
Chylomicron formation promotes intestinal absorption of full-length, antigenic OVA. Fasted mice were gavaged with 0.2 ml emulsions as described in Figure 1, except that ^125^I-OVA was replaced with 25 mg OVA. Blood samples were obtained from the submandibular vein at indicated time points and analyzed for OVA by Western blotting.

To test whether dietary LCT promote intestinal antigen absorption in a chylomicron-dependent manner, we gavaged fasted mice with ^125^I-OVA and [^3^H]-retinol as a chylomicron marker [22]–[24], together with either LCT, MCT, or LCT plus the inhibitor of chylomicron secretion, Pluronic L-81 (3% by volume). Kinetics experiments were performed to confirm that Pluronic L-81 effectively blocks intestinal absorption of retinol, and they revealed that peak retinol levels were obtained near 2 h after gavage (not shown). We therefore decided to analyze blood and MLN content of OVA and retinol at 90 minutes after gavage. As shown in Figure 1, dietary LCT significantly increased intestinal absorption of OVA into blood and MLN, compared with MCT, and the effect of LCT was blocked by Pluronic L-81. Retinol absorption mirrored OVA absorption (Figure 1), suggesting that OVA was absorbed and transported in a chylomicron-dependent fashion. Notably, OVA and retinol were both increasingly transported through the MLN, through which chylomicrons are transported. Figure 2B further illustrates the potency of Pluronic L-81 in its inhibiting effect of chylomicron secretion.

**Figure 3:**
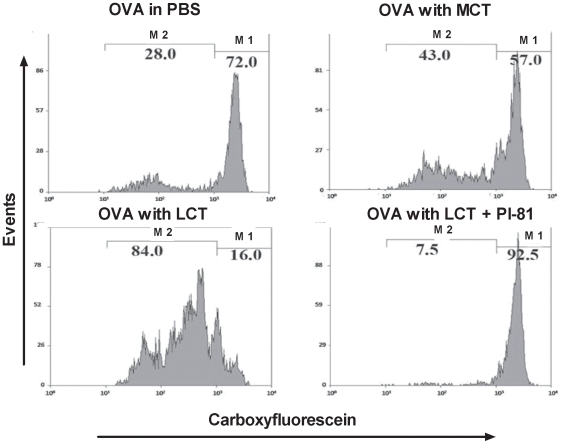
Chylomicron formation promotes systemic dissemination and antigen presentation of dietary antigen. Naïve BALB/C mice were injected with 2.5×10^6^ CFSE labeled T cells from DO11.10 TCR transgenic mice. After 24 h, the mice were fasted (4 h) and gavaged with OVA (25 mg) in 0.2 ml PBS or 25 mg OVA in 0.05 ml PBS+0.15 ml of either MCT, LCT, or LCT plus Pl-81. Mice were then fasted for an additional 6 h. Three days later, inguinal LN cells were isolated, stained with anti-CD4 and KJ1-26 (TCR clonotypic antibody), and analyzed by flow cytometry. Histograms show representative CFSE dilution profiles of gated CD4+, KJ1-26+ T cells as a measure of cell division. The % of cells under markers M1 and M2 represent cells which have not or have undergone cell division respectively. Each panel represents a typical result of three experimental repeats.

Since lack of LCT might lead to delayed OVA appearance in the plasma, we also performed a kinetics experiment, in which fasted mice were gavaged as in Figure 1, except that ^125^I-OVA was replaced with 25 mg unlabeled OVA. Plasma samples were obtained from the submandibular vein at 30, 60, 120 and 240 minutes after gavage, and analyzed for the presence of OVA by Western blotting. As shown in Figure 3, OVA levels were significantly higher in the LCT group at all time points, compared to mice gavaged with MCT or LCT+Pl-81.

Together, these data suggest that dietary LCT promote protein antigen absorption in a chylomicron-dependent manner.

### Chylomicron formation promotes systemic dissemination of dietary OVA

To determine whether the absorbed radioactivity represented antigenic OVA, and whether the absorbed antigen is systemically disseminated, we injected CFSE-labeled DO11.10 T-cells isolated from antigen-naive mice into antigen-naive BALB/C mice. After 24 h, the mice were fasted before receiving a single gavage with 25 mg OVA in 0.2 ml PBS, or emulsions of 0.05 ml PBS with 0.15 ml of either LCT, MCT, or LCT+Pluronic L-81. Food was withheld for another 6 hours. Two days later, inguinal lymph nodes were isolated, and T-cell proliferation was determined by measuring CFSE labeling intensity in CD4+KJ1-26+ cells. As shown in Figure 3, OVA gavage with LCT resulted in the most robust proliferation, which was completely blocked by Pluronic L-81. Control experiments in which mice were gavaged with similar emulsions without antigen did not show DO11.10 T-cell proliferation (not shown). Thus, chylomicron formation caused increased systemic dissemination of dietary OVA.

### Intestinally absorbed OVA is associated with plasma chylomicrons

**Figure 4:**
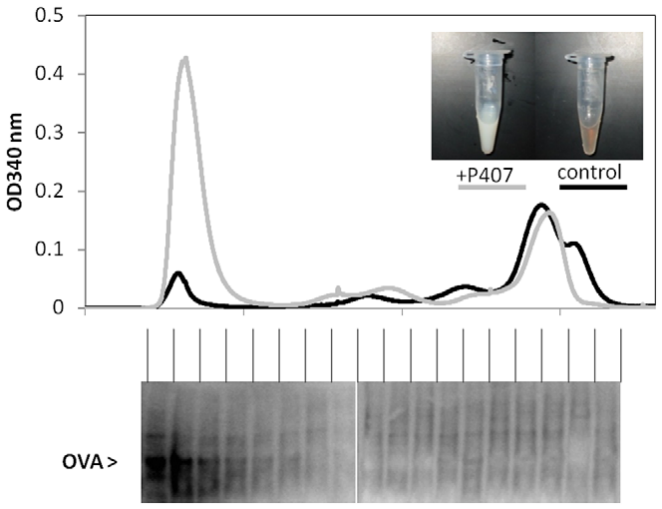
Plasma chylomicrons transport dietary OVA. Fasted mice were gavaged with 0.2 ml LCT-containing emulsions also containing 25 mg OVA. Plasma was isolated 1 h later, and 55 µl were fractionated via FPLC. The grey line of the chromatogram shows the elution profile of a mouse injected i.p. with Poloxamer P-407 1 h prior to gavage to inhibit chylomicron clearance, which caused a milky plasma appearance (inset) and a greatly increased first peak. The solid line shows the elution profile of a mouse not previously injected with Poloxamer P-407. The fractions of this mouse, indicated by the vertical separators, were subjected to immunoprecipitation for detection of OVA (lower panel).The experiment was repeated three times with similar outcomes.

The increase in systemic dissemination of dietary OVA as a result of chylomicron formation prompted us to test whether chylomicrons themselves were involved in transport, and hence dissemination, of dietary OVA. We therefore tested whether chylomicrons in the plasma are enriched with dietary OVA. Such an association between chylomicrons and OVA would not be unexpected given the emulsifying properties of this antigen [17], predicting affinity for oil-in-water emulsion particles such as chylomicrons. Fasted mice were gavaged with 25 mg OVA in 0.2 ml LCT/PBS emulsion, and plasma, collected 1 h later, was fractionated by FPLC. The chylomicron fraction eluted immediately after the void volume in the first peak, as illustrated by the fact that plasma from mice injected with the lipoprotein lipase inhibitor Poloxamer P-407 1 h before the gavage, which causes plasma chylomicron accumulation, yielded a much larger first peak (Figure 4) without affecting the height of other peaks. Fractions of the eluate obtained from non-Poloxamer treated mice were subsequently analyzed for the presence of OVA by immunoprecipitation. Interestingly, virtually all dietary OVA eluted with the chylomicrons in the first peak (Figure 4), suggesting that chylomicrons indeed transport dietary OVA. This could have accounted for systemic antigen dissemination, as observed in Figure 3.

### CaCo-2 cells secrete cell-associated OVA during chylomicron formation

**Figure 5:**
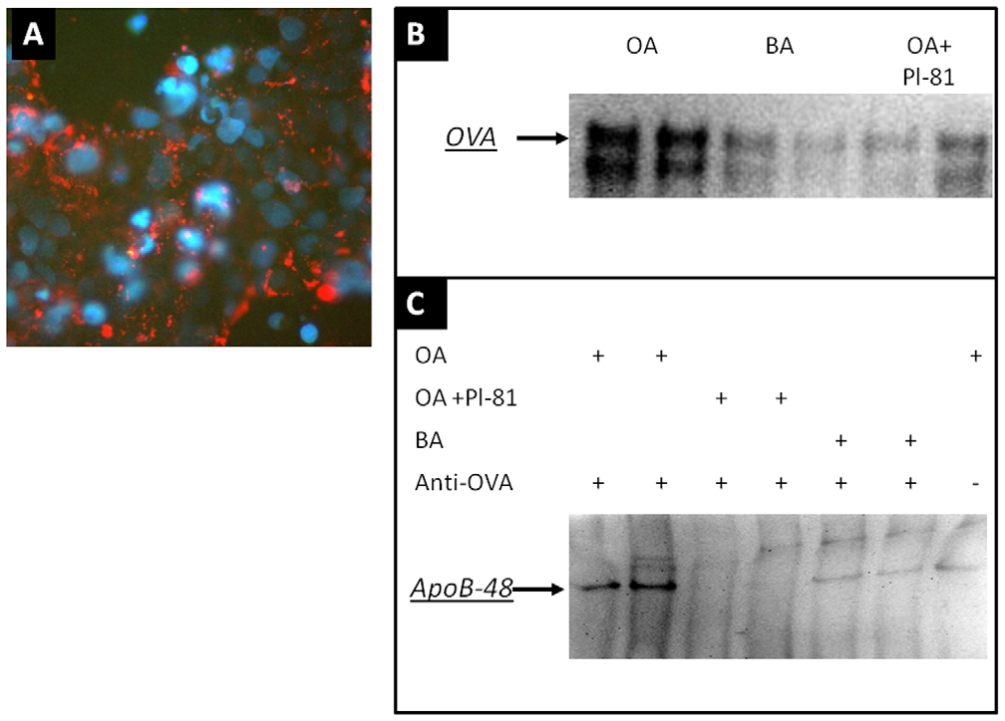
Uptake and secretion of OVA in association with chylomicrons by intestinal epithelial cells. (A) CaCo-2 cells were incubated with 20 µg/ml Alexa-red OVA for 1 h at 37°C. Nuclei were stained with DAPI (blue). (B) CaCo-2 cells on Transwell filters were incubated overnight at the apical side with 0.1 mg/ml OVA, washed from both sides, then incubated apically with 1.6 mM oleic acid (OA), butyric acid(BA), or oleic acid plus Pl-81 (2 µl/ml). Basolateral medium was collected 16 h later, and OVA was detected by immunoblotting. OVA was immunoprecipitated from the basolateral medium with anti-OVA coupled to protein A-Sepharose (or protein A-Sepharose only), followed by Western blotting of unwashed precipitate for detection of Apo-B. As shown in (C), ApoB-48 co-precipitated with OVA.

To explore how chylomicron formation promotes antigen absorption, we studied OVA secretion by CaCo-2 cells under conditions which preclude paracellular OVA leakage. First, however, CaCo-2 cells on glass slides were incubated for 1 h with 20 µg/ml Alexa-red OVA to determine OVA uptake. Fluorescent staining could be observed in association with the cell membrane and with vesicular structures (Figure 5A). Next, we determined whether chylomicron formation promotes basolateral release of OVA from CaCo-2 cells. Fully differentiated cells, grown for three weeks on Transwell membranes, were incubated overnight with 10 mg/ml OVA on the apical side. Unbound OVA was then thoroughly washed from both surfaces with serum-free medium, and the apical chamber received 0.5 mM taurocholate and 1.6 mM oleic acid, butyric acid, or oleic acid plus 0.2% Pl-81. After 16 h, the basolateral medium was collected and analyzed for OVA. As shown in Figure 5B, cells incubated with oleic acid secreted more OVA than cells incubated with butyric acid or with oleic acid plus Pl-81. To verify whether the secreted OVA was associated with chylomicrons, we performed immunoprecipitation with protein-A coupled to Sepharose, with or without prior addition of anti-OVA antibody. Strikingly, pull-down with anti-OVA resulted in significant pull-down of ApoB-48 (Figure 5C) from the oleic-acid groups. Pull-down with protein-A Sepharose lacking anti-OVA IgG led to limited (a-specific) pull down of ApoB-48. Thus, it appeared as if chylomicron secretion was associated with OVA secretion from IEC and, importantly, that the secreted OVA was associated with chylomicrons.

## Discussion

This study provides evidence for a novel role of dietary fat in the intestinal absorption of dietary protein antigens. We observed that dietary LCT promote absorption of OVA into MLN and blood, and that the effect of LCT is sensitive to inhibition of chylomicron secretion. We also observed that dietary OVA in plasma is mainly transported in association with chylomicrons, which likely mediates enhanced systemic dissemination of the antigen as reflected by increased proliferation of T-cells in peripheral lymph nodes not draining the intestine. Finally, we observed that cultured CaCo-2 IEC acquire OVA and partially secrete OVA during chylomicron formation. Moreover, the secreted OVA was associated with chylomicrons. We propose a novel mechanism, in which chylomicrons transport gut antigens from the intestinal epithelium via MLN to the circulation. This mechanism, which at least partially occurs through a novel mechanism of antigen secretion by IEC on newly formed chylomicrons, could have profound implications for immune responses to dietary antigens.

The mesenteric lymph drains the gastrointestinal tract, and mesenteric lymph nodes (MLN) play an important role in intestinal immunity, notably in the induction of oral tolerance [25]. Interestingly, after each meal, which almost always contains LCT, large amounts of chylomicrons are transported through mesenteric lymph and pass the MLN on their way to the thoracic duct where they spill over into the left-subclavean vein. Postprandial chylomicron transport thus leads to a typical milky appearance of the mesenteric duct. After each meal, antigen presenting cells and lymphocytes in the MLN are exposed to chylomicrons, which appear to stimulate mesenteric T-cell proliferation [10], [11]. This has typically been ascribed to mitogenic properties of free fatty acids [11], but we have previously demonstrated that dietary LCT also promote chylomicron-dependent absorption and transport of bacterial lipopolysaccharides (LPS) through the MLN [12]. This potent immune activator could perhaps contribute to postprandial T-cell activation.

In the present study we provide evidence that not only LPS from the intestinal microflora, but also T-cell antigens, in this case dietary OVA, are increasingly transported through the MLN and through the blood during chylomicron formation. Dietary LCT appeared to increase OVA absorption compared with MCT, which does not stimulate chylomicron production, and the effect of LCT was sensitive to the inhibitor of chylomicron secretion, Pluronic L-81. The fact that LCT enhanced absorption of OVA could theoretically be explained by a presumptive effect of long-chain fatty acids (oleic acid), liberated from triolein, on epithelial tight junctions. It has indeed been suggested that oleic acid may cause epithelial damage in vivo [26] and may cause paracellular leakage in CaCo-2 cells [27]. It cannot be entirely excluded, therefore, that LCT promoted intestinal OVA absorption by causing transient tight-junction leakage. Nevertheless, Pl-81 completely blocked the effect of LCT, and this detergent does not affect the uptake of fat into IEC, but rather prevents the secretion of chylomicrons [21], [28]. Since only fatty acids are taken up by IEC, and not triglycerides, this would suggest that Pluronic L-81 does not affect LCT hydrolysis in the gut, does not decrease fatty acid exposure of IEC, and therefore does not reduce any fatty-acid related stress that the IEC might experience. Moreover, in our CaCo-2 studies, we show preliminary evidence for an absorption mechanism that is independent of leakage, and occurs through secretion of OVA from the cell upon stimulation with oleic acid. Thus, we can likely rule out that Pl-81 decreased OVA absorption simply by decreasing fatty-acid mediated damage of IEC tight junctions.

We employed a single dose of LCT, and we do not how much LCT is minimally required for significant antigen appearance in the plasma. Taking into account the much higher rate of food intake in mice and extrapolating a mouse's bodyweight to that of an average person, the LCT dose was not necessarily unphysiological. Moreover, Pl-81 was able to completely block OVA absorption, even against the background of the seemingly robust dose of LCT. In any case, our observation that similar amounts of dietary MCT reduce antigen absorption at the very least suggests that MCT could modulate immune responses to dietary antigens by reducing antigen absorption.

Regardless of the absorption mechanism, our data suggest that chylomicrons profoundly affect the transport of dietary antigens through the body. Chylomicrons may act as vehicles for absorbed antigen, at least in case of OVA, as illustrated by the fact that virtually all of absorbed OVA in the plasma eluted in the same peak as the chylomicrons after FPLC analysis of postprandial blood. The affinity of chylomicrons for OVA is not unexpected given the emulsifying properties of OVA, which it shares with many other dietary allergens, such as peanut albumins [18] and milk casein [19]. We are currently testing whether other relevant dietary antigens also are transported through the blood in association with chylomicrons and how this affects immune responses to the antigens.

Macrophages are known to phagocytose chylomicrons [29], [30], and we hypothesize that this would facilitate antigen capture, analogous to what has been reported for lipid antigens [31]. Antigen transport in association with chylomicrons through the lamina propria and the MLN could thus facilitate antigen sampling by antigen presenting cells, which could perhaps play an important role in immune responses to the antigen. In this respect it is interesting that the MLN appears to be a critically important site for the induction of oral tolerance [25]. Furthermore, chylomicrons are the principal carriers of dietary retinol [22]–[24], and this vitamin is known to promote tolerogenic immune responses at the expense of pro-inflammatory Th17 responses [32]–[34]. Thus, chylomicrons would not only enhance antigen delivery, but also delivery of immune modulating substances, such as retinol and fatty acids. We have observed that chylomicron formation during antigen feeding indeed strongly affects immune responses to dietary antigens (unpublished observations).

Together, these studies reveal a novel role for dietary fat in the absorption and transport of dietary antigens. Unraveling this pathway may shed new light on immune responses to dietary antigens and may provide new insights into the important phenomena of oral tolerance and food allergies.

## Materials and Methods

### Materials

^125^I-OVA was prepared according to a slightly modified Iodine monochloride procedure [35], using grade V-OVA (Sigma-Aldrich). [^3^H]-retinol was purchased from American Radiolabeled Chemicals. MCT oil was purchased from Novartis, triolein (LCT), butyric acid, oleic acid and taurocholate from Sigma-Aldrich, and Pluronic L-81 (an inhibitor of chylomicron secretion [12], [21], [28] was a gift from BASF chemicals. Poloxamer P-407, a potent inhibitor of lipoprotein lipase [36], was purchased from Spectrum Chemical Manufacturing Corporation. Alexa-red OVA and carboxyfluorescein-succinimidyl ester (CFSE) were obtained from Invitrogen. Protein-A-sepharose and horseradish peroxidase-conjugated streptavidin were purchased from Sigma-Aldrich, biotinylated anti-OVA rabbit IgG from Abcam (ab8389), rabbit anti-OVA IgG from Millipore (ab1225), goat-anti Apolipoprotein B (Apo-B) from Calbiochem (178467), the KJ1-26 antibody from eBioscience (13–5808), and anti-mouse CD4 from BD-Pharmingen (553049).

### Cell Culture

CaCo-2 human IEC were purchased from ATTC and were cultured in 1∶1 DMEM:Ham's F12, supplemented with 5% fetal calf serum and penicillin/streptomycin/amphotericin (all from Hyclone). To study OVA uptake by CaCo-2 cells, the cells were grown on glass slides and incubated with 20 µg/ml Alexa-red OVA in complete medium for 1 h, fixed with 10% paraformaldehyde in PBS, and covered with DAPI-containing Vectashield (Vectorlabs) before being sealed with a glass coverslip. Cells were observed with an epifluorescence microscope (model BX50; Olympus) equipped with a cooled charge-coupled device camera. To study basolateral secretion of antigen and of chylomicrons, the cells were seeded in 12- well plates on top of Transwell filter inserts (Corning Transwell Clear; 3 µm pore size), and allowed to differentiate for 21 days [37]. To study OVA secretion, CaCo-2 cells were incubated overnight with 10 mg/ml OVA on the apical side and then washed from both surfaces with cold phosphate-buffered saline (PBS). Chylomicron formation was induced or prevented as described elsewhere [12], [37]. Briefly, cells were incubated from the apical side with 0.5 mM taurocholate and 1.6 mM oleic acid, with serum-free medium in the basolateral chamber. Control cells were incubated with butyric acid instead of oleic acid or with 2 µl Pl-81/ml. Chylomicron formation was estimated by sodium-dodecyl sulfate polyacrylamide gel electrophoresis (SDS-PAGE) under reducing conditions (no boiling) of freeze-dried basolateral medium and immunostaining of Apo-B [12]. The ∼250 kDa Apo-B signal represented the ApoB-48 isoform. OVA was detected by immunoblotting after SDS-PAGE (reduced and boiled samples).

### Mouse studies

Male BALB/C mice, 6 weeks old (Jackson Laboratory) were held in a room with a 12 h light 12 h dark cycle and were used at 8 weeks of age. The syngeneic DO11.10 mice were maintained as a breeding colony. This strain produces CD4 T-cells in which the majority expresses a transgenic T-cell receptor recognizing OVA peptide 323–339 presented on MHC-II [38]. The transgenic T-cell receptor binds the KJ1-26 antibody. Adoptive transfer experiments were carried out as described elsewhere [38], [39]. Briefly, splenocytes and lymph node cells from DO11.10 mice were labeled with CFSE and a cell suspension containing 2.5e6 CFSE-labeled DO11.10 CD4 T-cells was then injected into the tail vein of BALB/C recipients. Proliferation of KJ1-26+CD4+ T-cells in inguinal lymph nodes of the recipients was estimated by measuring CFSE staining intensity [40]. All animals were handled in strict accordance with good animal practice as defined by the relevant national and/or local animal welfare bodies, and all animal work was approved by the Institutional Animal Care and Use Committee of the University of Kentucky.

### Isolation of plasma chylomicrons and detection of associated OVA

Mouse plasma (55 µl) was fractionated by Fast Performance Liquid Chromatography (FPLC) using a BioRad BioLogic Duoflow system equipped with a Superose 6 column. The eluate was continuously monitored for absorption at 340 nm, and fractions were collected immediately after the void volume. The largest plasma aggregates, chylomicrons and some very low density lipoproteins (VLDL), elute in the first peak. To verify this, some plasma was analyzed from mice injected with the lipoprotein lipase inhibitor Poloxamer P-407 (1000 mg/kg) [36] 1 h before the gavage, which delays clearance of all triglyceride-rich lipoproteins, including chylomicrons. Low- and high density lipoproteins (LDL/HDL) elute later, whereas free proteins elute last. OVA in all fractions was detected by immunoprecipitation using protein-A Sepharose and rabbit anti-OVA IgG, followed by SDS-PAGE and immunoblotting with biotinylated anti-OVA IgG and streptavidin-HRP.
