# Correction: Chylomicrons Promote Intestinal Absorption and Systemic Dissemination of Dietary Antigen (Ovalbumin) in Mice

**DOI:** 10.1371/annotation/bc95caf3-62cf-4ecd-8a79-9116e62f4a50

**Published:** 2010-01-14

**Authors:** Yuehui Wang, Sarbani Ghoshal, Martin Ward, Willem de Villiers, Jerold Woodward, Erik Eckhardt

Shared authorship was inadvertently omitted for the first two authors. Yuehui Wang and Sarbani Ghoshal should be listed as co-first authors on this article.

